# Significant dose Escalation of Idarubicin in the treatment of aggressive Non- Hodgkin Lymphoma leads to increased hematotoxicity without improvement in efficacy in comparison to standard CHOEP-14: 9-year follow up results of the CIVEP trial of the DSHNHL

**DOI:** 10.1186/2193-1801-3-5

**Published:** 2014-01-03

**Authors:** Karin Hohloch, Carsten Zwick, Marita Ziepert, Dirk Hasenclever, Ulrich Kaiser, Andreas Engert, Heinz-Gert Höffkes, Frank Kroschinsky, Rolf Mesters, Andreas C Feller, Markus Löffler, Lorenz Trümper, Michael Pfreundschuh, On behalf of the the German High-Grade Non-Hodgkin´s Lymphoma Study Group (DSHNHL)

**Affiliations:** Department of Hematology and Oncology, Georg August University, 37099 Göttingen, Germany; Department Internal Medicine I, University of Saarland, 66421 Homburg/Saar, Germany; Institue for Medical Informatics, Statistics and Epidemiology, University of Leipzig, 04107 Leipzig, Germany; Department of Hematology, St Bernward Hospital, 31134 Hildesheim, Germany; Department of Internal Medicine I, University of Köln, 50937 Köln, Germany; Department of Hematology, City Hospital Fulda, 36043 Fulda, Germany; Department of Hematology and Oncology, University Hospital, 01307 Dresden, Germany; Department of Hematology and Oncology, University Hospital, 48149 Münster, Germany; Institute of Pathology, Medical University of Lübeck, 23538 Lübeck, Germany

**Keywords:** Aggressive B-cell lymphoma, Anthracycline, Dose escalation, Toxicity

## Abstract

**Background:**

Dose escalation and modification of CHOP has improved the prognosis of patients with aggressive lymphoma; even in the rituximab era, dose escalation for high-risk patients is exploited and frequently limited by drug toxicity. Idarubicin (Id) is a 4-demethoxy anthracycline analogue of daunorubicin with activity against lymphoma and has been reported to cause less cardiotoxicity than other anthracylines. The aim of this study was to replace doxorubicine with idarubicin in the CHOEP regimen and to find the maximum tolerable dose (MTD) of idarubicin based on hematotoxicity.

**Patients and methods:**

Between 11/96 and 09/98, 64 patients (pts) aged 18–75 yrs (pts. 18–60, LDH not elevated, >60 years all risk groups) with newly diagnosed aggressive lymphoma received 6 cycles of CIVEP-14 with an escalating dose of idarubicin, consisting of idarubicin (11–16 mg/m^2^ d1) and standard doses of cyclophosphamide, vincristine, etoposide, and prednisone with G-CSF support.

**Results:**

55 pts (median age 56 yrs) were evaluable for a final analysis with a median observation time of 9.3 years. The CR-rate was 77.4% ; the 5 and 8-year-EFS rates were 46.4% (95%CI 32.5-60.3%) and 43.5% (29.4-57.6%), respectively, and the 5- and 8 yr OS rates were 64.6% (51.7-77.5%) and 59.9% (46.4-73.4%). 14/55 patients have died due to lymphoma progression, and 2/55 patients (3.6%) due to treatment related toxicity, 4/55 due to other causes (3 infections, 1 acute heart failure). In a matched pair analysis comparing CHOEP-14 and CIVEP-14, CIVEP-14 had a higher hematotoxicity with no significant differences in the event free and overall survival for the two regimens.

**Conclusions:**

Thus, idarubicin cannot be used instead doxorubicin even if its dose is escalated to achieve similar hematotoxicity. Doxorubicin remains the standard anthracycline for the treatment of aggressive NHL.

**Electronic supplementary material:**

The online version of this article (doi:10.1186/2193-1801-3-5) contains supplementary material, which is available to authorized users.

## Introduction

Prior to the availability of the monoclonal CD20 antibody rituximab in the treatment of aggressive B cell non-Hodgkin`s lymphoma, the addition of etoposide (CHOEP)(Koppler et al. 1989) improved the outcome in young patients with good prognosis aggressive lymphoma and “dose-densified” CHOP, by reducing the treatment intervals from three to two weeks (CHOP-14), significantly improved the outcome in elderly patients above 60 years of age (Pfreundschuh et al. 2004b; Pfreundschuh et al. 2004a). However, even though etoposide improved outcome in younger patients, its use in elderly patients was compromised by toxicity and a subsequent decrease in dose intensity (Pfreundschuh et al. 2004a). Therefore, the identification of effective drugs without undue toxicity is – even in the era of immunotherapy and novel drugs – of clinical importance. Among the clinically more important side-effects of anthracyline administration is their cardiotoxicity, mainly manifesting itself by a decrease in cardiac ejection fraction and subsequent congestive heart failure, which can occurup to 25 years after drug administration; host-specific genetic factors have been shown to influence its frequency (Wojnowski et al. 2005). Idarubicin is an anthracycline with activity against lymphoma (Bonfante et al. 1983) with a longer half-life than doxorubicin. In preclinical studies idarubicin showed a better therapeutic index, particularly with regard to the cardiotoxicity rate. In a small single center study comparing CHOP with CIOP, the authors claimed to have achieved equivalent efficacy and lower toxicity for CIOP at a dose of 10 mg/m^2^ idarubicin (Zinzani et al. 1995). However, the idarubicin - doxorubicin dose equivalency had never been properly determined, nor had attempts been undertaken to employ the putatively superior toxicity profile in order to increase the idarubicin dose in an attempt to increase anti-lymphoma activity.

Therefore, the DSHNHL performed the CIVEP-14 trial that incorporated idarubicin instead of doxorubicin within the CHOEP-14 regimen in an attempt to further increase the intensity and efficacy of dose-dense chemotherapy without increasing toxicity. We hypothesized that in the study by Zinzani et al. doxorubicin had not been replaced with an equivalent (equitoxic) dose of idarubicin and that therefore efficacy could not be assessed correctly. We decided to use an equitoxic dose of CIVEP (compared to CHOEP) as the endpoint of the phase I/II trial. In addition, since continuous intravenous application of etoposide (Wilson et al. 1993) has been postulated to decrease toxicity in comparison to the standard bolus infusion, a prospective evaluation of c.i.v. etoposide was integrated into the CIVEP-trial. The aim of the CIVEP-14 phase I/II trial was therefore to determine the maximum tolerable dose (MTD) of idarubicin equitoxic to 50 mg/m^2^ doxorubicin by replacing doxorubicin with idarubicin in escalating dose levels in the standard CHOEP-14 regimen. A novel, modified Storer (Storer 1989) up-and-down algorithm was used to determine the MTD of idarubicin defined by pre-set criteria. At the highest idarubicin dose, etoposide application was switched from a bolus to a continuous intravenous application. We here report long-term follow up data with a median observation time for overall survival of 9 years. These data were subsequently compared with long-term follow-up data from the NHL B1/2 trials in a matched pair analysis. Even though the idarubicin dose could be escalated by 50%, our analysis shows a better long-term outcome with less toxicity for pts treated with standard CHOEP-14.

### Patients and methods

The study was conducted in accordance with the Declaration of Helsinki. The protocol was approved by the ethics review committee of Saarland University (coordinating center) and each participating center (see Additional file 1). All patients gave written informed consent. Patients were eligible if they had previously untreated, biopsy-confirmed, aggressive non-Hodgkin lymphoma according to the definition of the World Health Organization [WHO] classification (Jaffe et al. 2001). Young patients < = 60 years with low risk aggressive lymphoma (as defined by normal pretreatment LDH) and elderly patients aged 61–75 years with aggressive lymphoma of all risk groups were eligible.

Exclusion criteria included previous therapy including radiotherapy, bone marrow involvement > 25%, age < 18 or > 75 years, previously treated other malignancy, platelet counts (plt) < 100 000/mm^3^, white blood counts (WBC) < 3000/mm^3^, major organ dysfunction, known human immunodeficiency or active hepatitis B or C infection, WHO Performance status = 4, pregnancy and lactation. Patients with primary CNS lymphoma, mucosa associated lymphoid tissue lymphoma, lymphoblastic or Burkitt lymphomas were not included. Primary histopathological diagnoses were reviewed by a panel of six expert hematopathologists; reference pathology was available in 94.5% (52/55) of the cases.

### Staging

The stage of lymphoma was defined by means of physical examination, relevant laboratory parameters [complete blood count and blood chemistry including lactate dehydrogenase (LDH)], computed tomography of the chest, abdomen, bone marrow biopsy and other investigational procedures depending on clinical symptoms. Bulky disease was defined as the presence of a tumor mass with a maximal diameter ≥ 7.5 cm.

### Treatment

Patients received 6 cyles of CIVEP-14, consisting of cyclophosphamide, idarubicin, etoposide, vincristine and prednisolone, with 6 escalating dose levels for idarubicin as illustrated in Table 1. The I-16 patients received CIVEP with an idarubicin dose of 16 mg/m^2^ with etoposide as a bolus infusion in 10 and as a continuous 24-hr infusion in 14 patients. The results of an ancillary pharmakokinetic analysis for idarubicin and etoposide in a subset of seven pts from this trial have been published (Kroschinsky et al. 2004). Each patient was assigned to a given idarubicin dose level at registration. Dose levels were adjusted according to the up and down method according to Storer (Storer 1989) as described in detail in the statistics section. Granulocyte colony stimulating factor (G-CSF; filgrastim or lenograstim, doses according to the manufacturers’ recommendation) by daily s.c. administration was mandatory on days 5 to 13 of each cycle. Patients were to receive radiotherapy (36 Gy involved field irradiation) to sites of primary bulky disease and extranodal disease according to local standards.

**Table 1:** **CIVEP dose levels as described in Materials and Methods, 64 patients were assigned to different levels of Idarubicine according to a modified Storer up-and-down design**

CIVEP dose levels	I-11	I-12	I-13	I-14	I-15	I-16b	I-16c	Application
Cyclophosphamide	750	750	750	750	750	750	750	mg/m^2^ i.v. d1
Idarubicin	11	12	13	14	15	16	16	mg/m^2^ i.v. d1
Vincristine	2	2	2	2	2	2	2	mg (abs.) i.v.d1
Etoposide	100	100	100	100	100	100 bolus	100 civ	mg/m^2^ i.v d1 - 3
Prednisone	100	100	100	100	100	100	100	mg p.o. d1 – 5
G-CSF	300/480	300/480	300/480	300/480	300/480	300/480	300/480	μg/d s.c. d5 - 13
Patient# allocated excluded	1	3	4	12	20	10	14	
		1	1	2	2	2	1	

At dose level 16, patients were assigned to either bolus or continuous i.v. infusion of Etoposide.

### Assessment of toxicity and response

The WHO hematotoxicity grades were assessed for WBC, plt and hemoglobin (hgb) counts measured daily on chemotherapy days and every second day between cycles. Treatment was continued on day 15 if no relevant infection occurred, and if the total WBC count was > 2500 mm^3^ and plt were > 80 000 mm^3^.

All patients underwent restaging after 3 cycles of treatment and after the end of chemotherapy. Patients who received radiotherapy had an additional restaging 4 to 6 weeks after the end of radiotherapy. Restaging included the examination of all involved sites by appropriate methods. Tumor responses were classified as complete remission (CR), unconfirmed complete remission (CRu), partial remission (PR), stable disease, or progression under therapy according to the former International Workshop criteria(Cheson et al. 1999) with the modification that CR and CRu had to be confirmed by the first follow-up examination 2 months after restaging. Adverse events reported by the patient or observed by the treating physician were coded on the case report forms according to WHO grades. Follow up was carried out every 3 months in the first two years after treatment and every 6 months from the third up to the fifth year after treatment. Follow up for this evaluation was performed by phone interviews with the patients’ general practitioners.

### Statistical design, endpoints and statistical analysis

The aim of the CIVEP trial was to investigate the feasibility and toxicity of the CIVEP-regimen and to assess as primary endpoint the maximum tolerable dose of idarubicin in comparison to 50 mg/m^2^ doxorubicin. Secondary endpoints were remission rates, treatment-related mortality, event-free (EFS) and overall survival (OS). Using the Kaplan-Meier method, EFS and OS were calculated as the time from the beginning of therapy to the date of the first reported event, which was defined as either disease progression, initiation of additional (off-protocol) or salvage therapy, relapse, or death, whichever comes first for EFS and as death of any cause for OS. Patients with no reported event at the time of analysis were censored at the most recent assessment date.

The maximum tolerable dose (MTD) was defined as the dose level at which there was a 1/3 probability to experience a dose-limiting event averaged over the cycles 1–5. The following events were defined as dose-limiting (1) recovery of WBC (> 2500/mm^3^) later than day 15, (2) recovery of plt count (> 80.000/mm^3^) later than day 15, (3) active infection preventing start of next course. A generalization to parallel accrual of the up and down sampling scheme described by Storer et al. (Storer 1989) was developed to determine the idarubicin dose level for a new patient. The sampling algorithm made use of all the information on toxicities and dose-limiting events observed in the CIVEP cycles number one to five of patients who had been treated before. Essentially, a patient was assigned to the next higher dose level once two cycles, who had been treated at the current or a higher dose level, did not experience a dose-limiting event. The dose level for a new patient was decreased by one step if a patient experienced a dose-limiting event during a cycle at the current or a lower dose level. Toxicity results that did not have an immediate effect on the current dose by these rules were queued to have an effect in case a change in the current dose level rendered them relevant. Using this sampling scheme the current dose level oscillates stochastically around the maximal tolerable dose. A close telephone monitoring after each of the chemotherapy courses was necessary to gather the information from treating physicians whether the retreatment on day 16 was possible. If this was not the case, the patient dose level had to be reduced for the following cycles by one dose level.

Simulations of the dose-finding algorithm had shown that about 40 patients had to be included, and another 10 pts had to be included for the etoposide c.i. vs i.v. comparison.

In order to compare efficacy and toxicity data, CIVEPpts and pts treated with CHOEP-14 in the NHL-B1/B2 trials [4,5] were matched with regard to the IPI factors in a 1:1 ratio. For each of the CIVEP pts the pattern of IPI factors was assessed, and a pt from the NHL-B1/B2 trial with the same pattern was selected at random. Hematotoxicity, side effects and therapeutic interventions were compared by chi square tests and Fisher exact tests if required. Log rank tests were performed to test the EFS and OS between CIVEP and CHOEP treatment. The significance level was p = 0.050. Statistical analyses were done with SPSS 15.0.

### Patient characteristics

From November 1996 to September 1998, 64 patients were enrolled at 7 participating institutions (see Additional file 1). Dose level assignment is shown in Table 1. Nine patients (one patient in dose level I-12 and I-13, 2 patients in dose level I-14 and I-15, and 2/1 patients in dose level I-16b/c) were excluded from the final analysis for the following reasons: diagnosis according to inclusion criteria not confirmed by reference pathology (6 pts.), elevated LDH in a young patient < = 60 years (1 pt.), previously treated other malignancy (1 pts.), leaving 55 patients for evaluation (Table 2). Median age was 56 years (range 23–71 years), 36.4% of patients were > 60 years. Lymphoma histology showed 38 patients to have diffuse large B- cell lymphoma, 7 follicular lymphoma grade III, 3 NOS and 2 patients unclassified B cell lymphoma, 1 PTCL, 3 without material and 1 with material, but technically insufficient . Most patients had an IPI 0 or 1 (70.9%), 12 patients had bulky disease, 14 B symptoms and 31 patients had extranodal involvement (Table 2). The patient characteristics for the matched NHL-B1/B2 population is also shown in Table 2.

**Table 2:** **Patient characteristics**

Regimen	6xCIVEP-14	6xCHOEP-14
	n=55	n=55
	n (%)	n (%)
Male	28 (50.9%)	33 (60.0%)
Female	27 (49.1%)	22 (40.0%)
Age years median (range)	56 (23-71)	56 (25-73)
> 60 years	20 (36.4%)	20 (36.4%)
Histologies WHO classification		
B-cell:		
Diffuse large B-cell lymphoma	38	38
Follicular lymphoma III°/	7	6
Follicular lymphoma III° + DLBCL		
NOS	3	7
Unclassified (insufficient mat.)	2	3
T-cell:		
PTCL	1	1
No material/unclassified:	3/1	-
IPI		
0,1	39(70.9%)	39 (70.9%)
2	11 (20.0%)	11 (20.0%)
3	2 (3.6%)	2 (3.6%)
4,5	3 (5.5%)	3 (5.5%)
Bulky disease	12 (21.8%)	13 (23.6%)
B-symptoms	14 (25.5%)	15 (27.3%)
Extranodal involvement	31 (56.4%)	31 (56.4%)

In a matched pair analysis, pts recruited to CIVEP-14 and CHOEP-14 from the previously published NHL-B1/B2 trials were compared.

## Results

### Treatment

Of the 55 pts eligible for the final evaluation, 34 pts completed treatment according to protocol. 21 pts did not complete the treatment protocol due to insufficient response (5 pts; 1 PR, 1 NC, 3 PD), excessive toxicity (8 pts), major protocol violations (5 pts), comorbidity (2 pts) and withdrawal of therapy by the patients wish (1 pt). The dose levels I-11, I-12 and I-13 were applied without idarubicin dose deescalation. At the next dose level I-14 was deescalated to I-13 for one pt, the I-15 dose level to I-14 for 7 pt. The final dose level of I-16 was reached after 40 pts. The remaining patients were treated with an idarubicin dose of 16 mg with an etoposide 24 h c.i.v. (14 pts) or bolus (10 pts) application. 11 Patients received the optional pre-phase treatment with prednisolone (100 mg day 1–7) and vincristine (2 mg, day 1).

**Figure 1:**
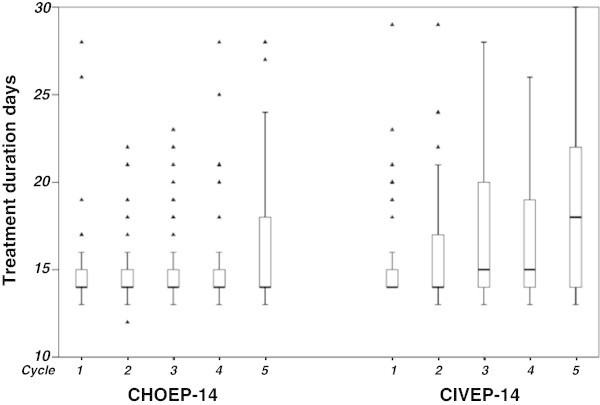
**Treatment duration for cycle 1 to 5 for CHOEP-14 and CIVEP-14.**

**Figure 2:**
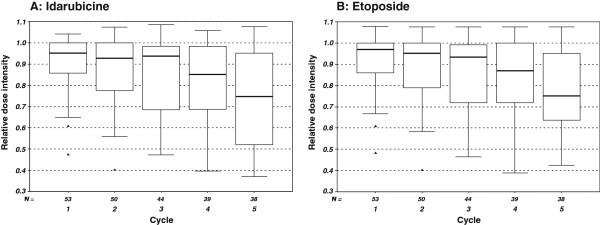
**Relative dose intensities of idarubicine (A) and etoposide (B) during cycles 1 to 5.** Relative dose intensities: actually applied doses/planned doses in relation to time/intended times are given in boxplots (upper limit of box: upper quartile, line within box: median, lower limit of box: lower quartile, whisker: last value within one and a half box lengths, more distant values are outliers).

The relative dose for etoposide was 100.0% for each of the cycles and for idarubicin between 93.2% and 99.3%. The median duration of cycle intervals increased from cycle one and two with a cycle duration of 14 days to 15 days in cycle 3 and 4 to 18 days in cycle 5, due to hematotoxicity and a consecutive delay of subsequent cycles as defined in the study protocol. In comparison the median duration of cycle intervals was 14 days over all cycles for the matched patients from NHL-B1/B2 trial treated with CHOEP-14 (Figure 1). Due to treatment delays related to excessive toxicities in the CIVEP trial, the relative dose intensities for etoposide and idarubicin showed a decrease from cycle one to six (Figure 2). G-CSF (filgrastim) was given for a median duration of 9 days through all planned six treatment cycles. In total, 4 chemotherapy administrations were given without G-CSF, for 10 administrations the duration of G-CSF application was not documented.

### Toxicity

Cardiac toxicity was assessed by echocardiography before and after chemotherapy and radiotherapy in all patients. There were no significant differences (p = 0.766) in the fractional shortening rate before (median: 35.5) and after treatment (median: 35) at all dose levels. Grade III/IV cardiotoxicity was also not significantly different in the two trials (Table 3).

**Table 3:** **Side-effects and therapeutic interventions according to regimen Side effects according to the WHO scale were compared in a matched pair analysis**

Effects	6xCIVEP-14	6xCHOEP-14	p-vlaue
Hematotoxicty*	% pts.	% pts.	
Leukocytopenia (<1*10³/mm³)	80.0	39.1	<0.001
Thrombocytopenia (<50*10³/mm³)	57.5	31.6	=0.021
(<8 g/dl)	74.5	35.8	<0.001
Side effects **WHO Grade III-IV**			
Alopecia	61.8	67.9	n.s.
Infection	14.5	11.1	n.s.
ANE	10.9	5.5	n.s.
Polyneuropathy	7.3	3.7	n.s.
Pulmonary toxicity	7.3	3.7	n.s.
Cardiotoxicity	5.5	3.7	n.s.
Renal toxicity	0.0	1.9	n.s.
Therapeutic interventions (per patient)	%	%	
Red blood transfusion	69.1	38.2	=0.001
Platelet transfusion	18.2	5.5	=0.039
Antibiotics	54.5	41.8	=0.182

ANE: acute nausea and vomiting, n.s. not significant.

Values in the table represent the percentage of patients experiencing the respective side effect at least once. based on blood values from nadir intervals for leukocytopenia/thrombocytopenia: day 9-11/11-13 (CIVEP-14) and day 8-10/10-12 (CHOEP-14).

Hematotoxicity was significantly more pronounced in the CIVEP-14 cohort in comparison to patients from the NHL-B1/2 trials.

Hematotoxicity was assessed by serial blood counts during treatment (daily) and treatment intervals (every 2^nd^ or 3^rd^ day). Compared to CHOEP-14, hematotoxicity was significantly higher in CIVEP-14. Grade IV° leukopenia occurred in 80.0%, thrombocytopenia grade III/IV° in 57.5% and anemia grade III/IV° in 74.5% of the CIVEP-14 treated patients, compared to 39.1%, 31.6% and 35.8% in the CHOEP-14 treated patients respectively. In comparison to the published data of NHL B1/B2, therapeutic interventions in the CIVEP-14 were more pronounced regarding red blood cell and platelet transfusions and there was a trend to more administration of antibiotics (Table 3). Therefore, dose escalation to I-16 resulted in more than equitoxic hematotoxicity in patients treated with CIVEP-14.

### Response and survival

CR rate after the end of treatment was 77.4% (95% CI 66.1-88.7%), with 41 patients reaching a CR. 5 patients reached a PR, one patient had a NC, 6 patients showed progressive disease. For 2 patients outcome is not known.

**Figure 3:**
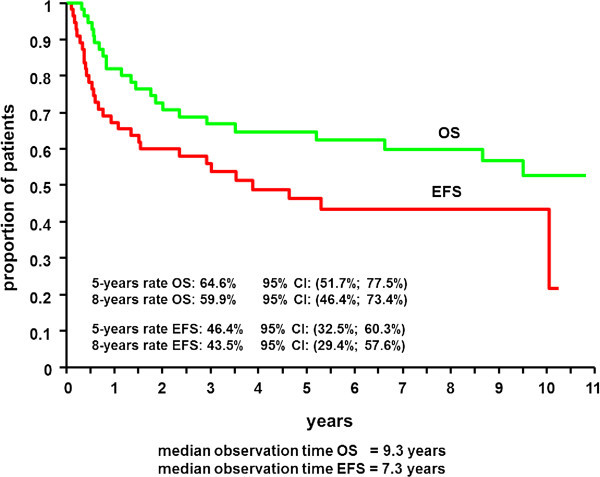
**Event free (EFS) and overall survival (OS) of CIVEP -14 (n=55).** The proportion of patients with event free and overall survival are depicted, with median observation times of 9.3 years for OS and 7.3 years for event free survival.

**Figure 4:**
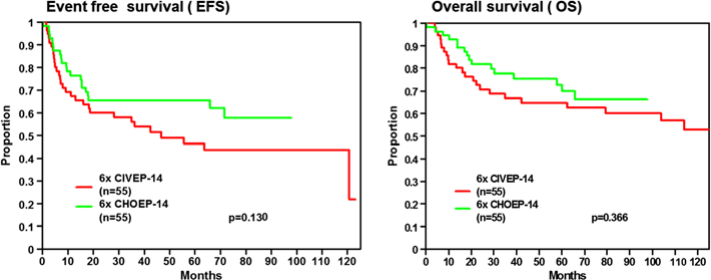
**Event free survival and overall survival: Comparison of CIVEP-14 and CHOEP-14.** In a matched pair analysis, EFS and OS were compared for patient cohorts treated in the CIVEP-14 trial and suitably matched patients from the previously published NHL B1/B2 trials. The median observation time for the CHOEP-14 group for EFS/ OS are 5.5/5.3 years. There is no statistically significant difference in outcome, with a trend towards better outcome with the standard CHOEP-14 regimen.

At a median observation time of 9.3 years for overall survival and 7.3 years for the event free survival the 5- and 8-year OS rates were 64.6% (95% CI 51.7-77.5%) and 59.9% (95% CI 46.4-73.4%) the 5 and 8 year EFS rates were 46.4% (95% CI 32.5-60.3%) and 43.5% (95% CI 29.4-57.6%), respectively (Figure 3). In comparison to CHOEP-14 (Table 1), EFS and OS did not show a significant superiority for CIVEP-14 (Figure 4) despite higher toxicity.

23 pts (41.8%) have died at the time of this final analysis with a median observation of 9.3 yrs for OS. Treatment related mortality was observed in two patients 3.6% (acute toxicity). 14 (25.5%) pts. have died due to lymphoma progression, 4 patients (7.3%) due to comorbidities, three of them due to infections, one because of acute cardiac failure. For 3 pts the cause of death is unknown.

## Discussion

Aggressive B cell lymphoma can be cured by chemotherapy alone, as demonstrated by the pivotal SWOG trial (Fisher et al. 1993). Fisher and colleagues also showed that dose escalation does not necessarily lead to improved outcome due to increased toxicity. Subsequent trials in the pre-rituximab era, however, supported the concept of moderate dose intensification and dose density (Coiffier 1995; Zwick et al. 2011; Pfreundschuh et al. 2004b), also in the elderly (Bastion et al. 1997; Pfreundschuh 2010). Initial observations in the rituximab era suggested that rituximab “equalizes” the positive effect of dose escalation (Pfreundschuh et al. 2006); recently, however, interest in dose escalation has been renewed based on several trials(Recher et al. 2011; Schmitz et al. 2006). Therefore, strategies are sought especially in elderly patients to increase or maintain dose intensity without undue toxicity. Exchanging doxorubicin with a putatively less toxic and equally effective anthracyline is a commonly applied strategy (Corazzelli et al. 2011)even though direct comparisons between “substitutes” and standard doxorubicin are often lacking. Idarubicin was first introduced in the treatment of lymphoma as a monotherapy showed promising activity and low toxicity, especially cardiotoxicty (Coonley et al. 1983; Errante et al. 1991; Case et al. 1992). In 1994 in a phase II study Zinzani et al. (Zinzani et al. 1995) claimed that CIOP, a regimen containing 10 mg/m^2^ idarubicin instead of 50 mg/m^2^ doxorubicin, was equivalent to CHOP regarding remission rates and overall survival; toxicity was, even slightly reduced in the CIOP arm compared to the CHOP treated patients. Often, small phase II trials with short follow-up and possible selection bias lead to overly optimistic conclusions regarding the efficacy and toxicity of novel substances. We therefore decided to compare the results of a trial replacing doxorubicin with adequately (and escalating) doses of idarubicin (CIVEP-14) that was conducted from 1996–1998 in the pre-rituximab era in a matched pair analysis with its parent regimen, CHOEP-14. The CIVEP phase I/II trial had been designed in 1996 to determine the MTD of idarubicin utilizing a generalization of the Storer up-and-down design. This algorithm allows a rapid and at the same time safe dose escalation in a phase I design, provided, timely reports of toxicities in allocated patients are entered into the trial data base. Since these toxicities were collected by phone on a weekly basis in this trial, the final dose level could be reached after very few patients, permitting a sufficient accrual of patients at the final dose level in order to collect information for the secondary efficacy endpoints. We show here that the idarubicin dose could safely be increased by 50% to 16 mg/m^2^, whereas clinical phase II trials at that time had preferred a dose of 10 mg/m^2^ (Zinzani et al. 1995).

Long-term follow-up demonstrated that the substitution of doxorubicin by dose escalated idarubicin is indeed feasible and safe. The matched-pair analysis with CHOEP-14 treated patients clearly demonstrated a significantly increased hematotoxicity with no visible advantage in EFS or OS. As idarubicin, as shown before in an ancillary pharmacokinetic and in vitro study to this trial (Kroschinsky et al. 2004), seems to have a higher stem cell toxicity in comparison to other anthracyclines, its use in hematological oncology will remain limited to acute myeloid leukemia. A BNLI phase III trial published in 2005 by Burton et al. (Burton et al. 2005) also showed a significant reduction in the CR rate translating into a reduced PFS comparing CHOP with CIOP at the 10 mg level in a younger good-prognosis lymphoma population. For both the Zinzani and Burton trials we conclude that the putatively superior toxicity profile was due to an underdosing by more than 50% in comparison to doxorubicine. Substituting long-standing active drugs in lymphoma regimens demands a careful assessment of equally effective doses. As in the idarubicin case, this can actually translate into significantly higher toxicities.

This trial was designed before the introduction of rituximab into the treatment of aggressive B-cell lymphomas (Feugier et al. 2005). So far, data on rituximab-containing idarubicin combinations have not been published. However, it is safe to assume that, such data would not differ significantly from our CIVEP data. As dose escalation especially in high-risk populations has recently regained strategic significance in DLBCL, doxorubicin as an important component of the CHOP regimen should not be replaced by other anthracyline drugs based on uncontrolled phase II trials. Matched pair analyses with sufficient patient numbers such as the one presented here may alleviate the need for randomized trials under certain circumstances.

In conclusion, we have presented long-term follow up over more than 9 years with a modified CHOEP regimen in aggressive lymphoma that clearly demonstrates the curative potential of this CIVEP regimen without undue late toxicity. We also convincingly demonstrate in a large matched-pair analysis of more than 100 patients that doxorubicin remains the anthracycline of choice in aggressive lymphoma as idarubicin adds hematotoxicity, but does not increase efficacy.

## Electronic supplementary material

Additional file 1: **Participating Institutions (n=7).** (DOCX 22 KB)
