# Supplementary material for: Significant dose Escalation of Idarubicin in the treatment of aggressive Non- Hodgkin Lymphoma leads to increased hematotoxicity without improvement in efficacy in comparison to standard CHOEP-14: 9-year follow up results of the CIVEP trial of the DSHNHL
Source: Springerplus. 2014 Jan 3;3:5. doi: 10.1186/2193-1801-3-5 (PMC3890437; doi:10.1186/2193-1801-3-5)
Supplement: Supplementary file 1 — Additional file 1: Participating Institutions (n=7). (DOCX 22 KB) [file 40064_2013_763_MOESM1_ESM.docx]

**Appendix**

**Participating Institutions (n=7)**

Homburg (Med. Universitätsklinik Homburg) n = 22

Marburg (Universitätsklinikum Marburg) n = 10

Köln (Universitätsklinik Köln) n = 9

Magdeburg (Medizinische Akademie Magdeburg) n = 8

Dresden (Universitätsklinikum Dresden) n = 7

Münster (Med. Universitätsklinik Münster) n = 7

Chemnitz (Krankenhaus Küchwald Chemnitz) n = 1
